# Immune resistance in hepatobiliary and pancreatic cancers: rethinking the tumor microenvironment beyond checkpoint blockade

**DOI:** 10.3389/fimmu.2026.1870326

**Published:** 2026-07-15

**Authors:** Jingyi Xu, Lei Yang, Shuang Wang, Liusheng Wu, Yuehua Liang, Xialin Xie, Wenqiang Wang, Lu Gao, Kai Sun, Jun Yan

**Affiliations:** 1 Department of Liver Surgery, Beijing Tsinghua Changgung Hospital, School of Clinical Medicine, Tsinghua Medicine, Tsinghua University, Beijing, China; 2 Department of Basic Medicine, Beijing Health Vocational College, Beijing, China; 3 School of Biomedical Engineering, Tsinghua University, Beijing, China

**Keywords:** biliary tract cancer, checkpoint blockade, hepatocellular carcinoma, immune resistance, immunotherapy, pancreatic ductal adenocarcinoma, tumor microenvironment

## 1 Introduction

Immune checkpoint inhibitors (ICIs) have reshaped the treatment landscape of several solid tumors, yet their clinical impact across hepatobiliary and pancreatic cancers remains uneven. Hepatocellular carcinoma (HCC) typically arises in a chronically inflamed, tolerogenic liver where antiviral immunity, metabolic injury, fibrosis, angiogenesis, and immune suppression converge (1, 2). In advanced HCC, atezolizumab plus bevacizumab and tremelimumab plus durvalumab have established immunotherapy-based first-line options (3–6). In biliary tract cancer (BTC), adding durvalumab or pembrolizumab to gemcitabine-cisplatin improves survival, although benefit remains limited for many patients (7–10). BTC is biologically heterogeneous, with desmoplastic stroma, macrophage-rich inflammation, cancer-associated fibroblasts, and variable antigen presentation shaping the immune contexture (11–13). By contrast, pancreatic ductal adenocarcinoma (PDAC) remains largely refractory to ICI-based therapy, except for rare microsatellite instability-high or mismatch repair-deficient tumors (14–21).

This heterogeneity should not be interpreted as a simple hierarchy of tumor “immunogenicity”. Rather, it reflects the fact that HCC, BTC, and PDAC are governed by distinct tumor microenvironmental constraints. In many patients, PD-1/PD-L1 signaling is not the dominant rate-limiting step. The decisive barriers may lie upstream, at the level of antigen presentation and T-cell priming, or downstream, at the level of T-cell trafficking, stromal exclusion, myeloid suppression, vascular dysfunction, and metabolic competition. We therefore argue that immune resistance in hepatobiliary and pancreatic cancers should be reframed as a tumor microenvironment-centered problem rather than a checkpoint-centered problem alone.

## 2 Checkpoint blockade is insufficient when the cancer-immunity cycle is interrupted

Checkpoint blockade requires a partially intact cancer-immunity cycle. Tumor antigens must be released and presented, dendritic cells must initiate productive T-cell priming, effector T cells must traffic into tumor nests, and cytotoxic lymphocytes must retain function within hostile tissue ecosystems. In hepatobiliary and pancreatic cancers, one or more of these steps is frequently impaired.

Failure of single- or dual-agent checkpoint blockade does not mean a tumor is immunologically silent. Many tumors are immunologically active but misdirected, spatially excluded, metabolically exhausted, or suppressed by stromal and myeloid networks. Escalating checkpoint blockade may therefore add toxicity without resolving the dominant barrier. If priming is inadequate, few effectors can be reinvigorated; if T cells remain outside malignant glands, cytotoxicity cannot reach tumor cells; and if macrophages, myeloid-derived suppressor cells, or CAFs dominate, suppression may persist despite PD-1/PD-L1 blockade.

Accordingly, future strategies should identify the barrier that prevents immune execution and pair ICIs with a corrective intervention: priming for absent effectors, access-restoring therapy for excluded T cells, suppressive-network reprogramming for myeloid/CAF-dominant tumors, and vascular or metabolic remodeling for hostile tissue states.

## 3 Distinct immune ecologies across HCC, BTC, and PDAC

HCC illustrates inflammation without effective immunity. Chronic hepatitis, alcohol-related injury, metabolic dysfunction, and cirrhosis create persistent immune activation in a tolerogenic liver. Exhausted CD8^+^ T cells, regulatory T cells, tumor-associated macrophages, myeloid-derived suppressor cells, and VEGF-driven abnormal angiogenesis restrain antitumor immunity (1, 2). Atezolizumab plus bevacizumab is mechanistically informative because it blocks PD-L1 while targeting VEGF-mediated vascular and immune suppression (3, 4). The durable signal with tremelimumab plus durvalumab supports broader immune reactivation in selected HCC subgroups (5, 6). Etiology also matters because viral hepatitis, alcohol-related liver disease, and metabolic dysfunction-associated steatotic liver disease may influence immune-cell composition, antigen presentation, and systemic therapy response.

BTC is immunologically heterogeneous. Intrahepatic cholangiocarcinoma, extrahepatic cholangiocarcinoma, and gallbladder cancer differ anatomically, molecularly, and immunologically. Many BTCs nevertheless share a desmoplastic, macrophage-rich, fibroblast-enriched microenvironment with heterogeneous antigen presentation and spatially variable T-cell states (11–13). TOPAZ-1 and KEYNOTE-966 show that chemoimmunotherapy improves outcomes in advanced BTC (7–10), but not that PD-1/PD-L1 blockade alone is sufficient. Chemotherapy may increase antigen release and transiently reshape immune suppression and tumor-stroma interactions, while modest response depth suggests first-line chemoimmunotherapy only partially addresses BTC immune resistance.

PDAC represents the most stringent example of microenvironment-driven immune resistance. Dense fibrotic stroma, poor perfusion, hypoxia, cancer-associated fibroblast activity, suppressive myeloid infiltration, and weak dendritic-cell priming produce immune exclusion rather than simple immune absence (14–17). The poor activity of durvalumab with or without tremelimumab in metastatic PDAC was therefore biologically predictable (18). PDAC is resistant because antitumor immunity is prevented from becoming spatially, functionally, and metabolically effective. The responsiveness of MSI-H/dMMR tumors to pembrolizumab across solid tumors reinforces that when antigenicity and immune recognition are strong, even traditionally resistant sites may respond (19–21). The challenge is to generate, recruit, and sustain effective immune effectors within a hostile stromal niche.

## 4 Four dominant barrier domains

**Figure 1:**
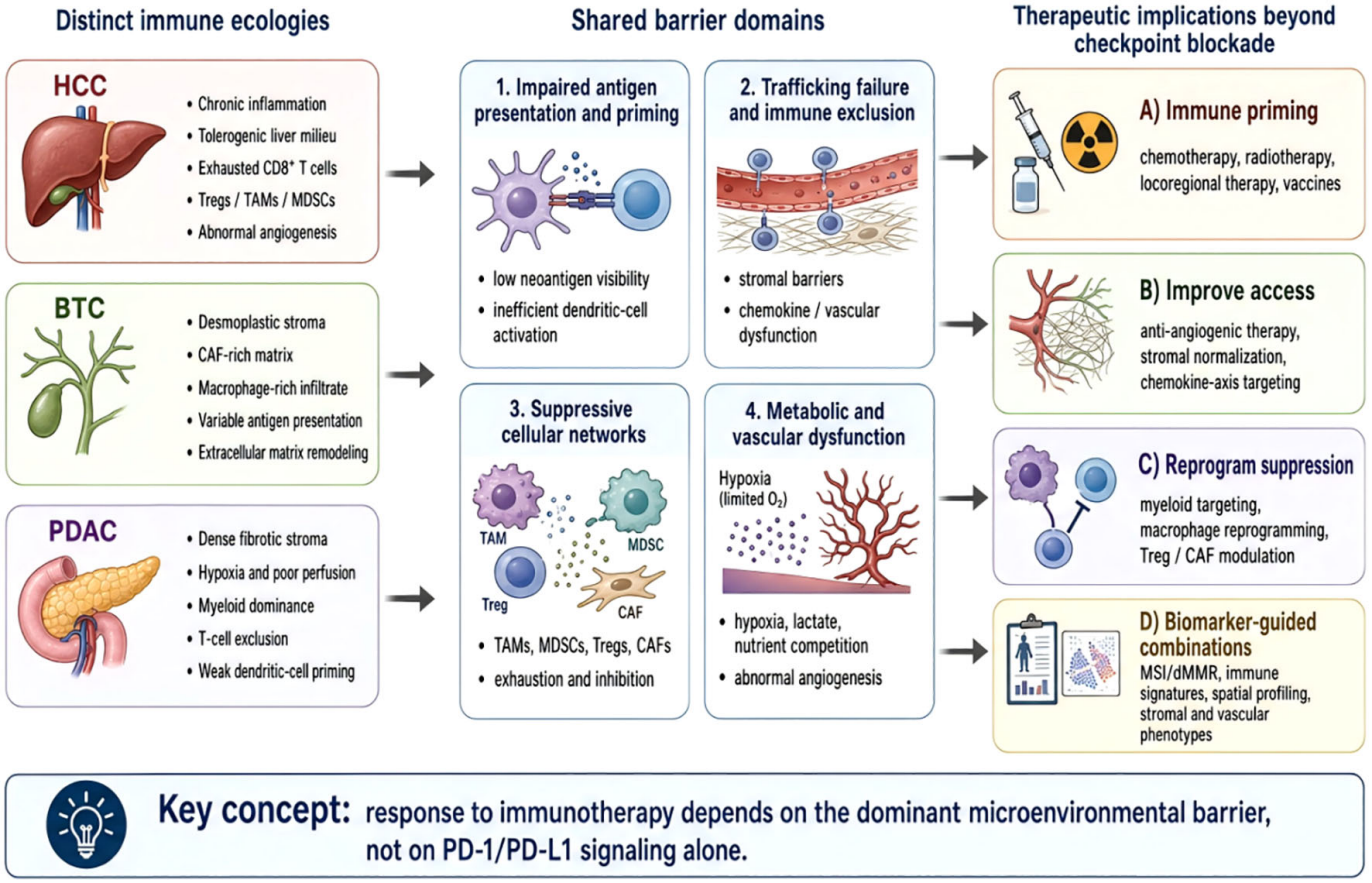
A tumor microenvironment-centered framework of immune resistance in hepatobiliary and pancreatic cancers.

A tumor microenvironment-centered framework can classify immune resistance into four overlapping barrier domains (**Figure 1**).

These domains are not independent. VEGF-driven vascular abnormality can simultaneously restrict T-cell entry, intensify hypoxia, and recruit myeloid cells; CAF-derived TGF-beta/CXCL12 can link exclusion with macrophage/Treg accumulation. Therefore, the practical task is to identify the dominant reversible bottleneck, not assign mutually exclusive categories.

This schematic summarizes representative immune microenvironment features and dominant resistance barriers across HCC, BTC, and PDAC. HCC commonly develops in a chronically inflamed but tolerogenic liver milieu, characterized by exhausted CD8^+^ T cells, regulatory T cells, tumor-associated macrophages, myeloid-derived suppressor cells, and abnormal angiogenesis. BTC frequently exhibits a desmoplastic and cancer-associated fibroblast-rich matrix, macrophage-rich infiltration, extracellular matrix remodeling, and heterogeneous antigen presentation. PDAC is marked by dense fibrotic stroma, hypoxia, poor perfusion, myeloid dominance, T-cell exclusion, and weak dendritic-cell priming. These disease-specific immune ecologies converge into four shared resistance domains: impaired antigen presentation and priming, trafficking failure and immune exclusion, suppressive cellular networks, and metabolic or vascular dysfunction. Therapeutic implications beyond checkpoint blockade include immune priming strategies, improved immune-cell access, reprogramming of suppressive networks, and biomarker-guided combination therapy. This figure is intended as a conceptual framework rather than a universal description of every tumor, and it should be interpreted together with tumor-cell-intrinsic and host-factor domains discussed below.

First, some tumors are limited by impaired antigen presentation and insufficient T-cell priming. This may reflect low antigenicity, defective antigen-processing machinery, poor dendritic-cell recruitment, or dysfunctional maturation. Dendritic cells generate and sustain T-cell-inflamed tumor microenvironments, and their impairment can blunt checkpoint blockade even when PD-1/PD-L1 signaling is present (22, 23). In these tumors, immune priming strategies, including chemotherapy, radiotherapy, locoregional therapy, and vaccines, may be more relevant than checkpoint escalation alone. In HCC, locoregional and systemic therapies can reshape the immune microenvironment, depending on treatment type, timing, tumor burden, and background liver disease (24). Thus, local tumor injury may provide antigenic and inflammatory signals, but requires rational immunological integration rather than empiric ICI addition.

Second, tumors may be dominated by trafficking failure and immune exclusion. This is particularly relevant in PDAC and subsets of BTC, where stromal barriers, cancer-associated fibroblasts, and chemokine gradients prevent effector cells from reaching malignant epithelial compartments (25–27). In HCC, abnormal angiogenesis can similarly compromise immune-cell entry and function. Therapeutically, this argues for normalizing rather than indiscriminately ablating the microenvironment. Anti-angiogenic therapy, stromal modulation, and chemokine-axis targeting should be understood as immune-access strategies. The objective is to restore spatial contact between cytotoxic lymphocytes and malignant cells, not simply to reduce stromal mass.

Third, suppressive cellular networks can actively disable antitumor immunity. Tumor-associated macrophages, myeloid-derived suppressor cells, regulatory T cells, and cancer-associated fibroblasts may reinforce one another through cytokines, chemokines, metabolic products, and cell-cell contact. In such cases, the clinical question is not simply whether PD-1 blockade should be added, but whether myeloid targeting, macrophage reprogramming, Treg modulation, or CAF-directed strategies are needed to convert a suppressive immune ecology into a permissive one. This issue is especially relevant in BTC and PDAC, where myeloid and fibroblast programs may dominate over lymphocyte-inflamed patterns.

Fourth, metabolic and vascular dysfunction can impose a non-redundant layer of immune resistance. Hypoxia, lactate accumulation, nutrient competition, and abnormal perfusion reduce T-cell fitness and may amplify myeloid suppression. This is especially relevant in PDAC and HCC, but also applies to desmoplastic BTC. The clinical success of anti-VEGF-containing immunotherapy in HCC provides proof of principle that remodeling the ecological conditions of the tumor can enhance the effect of checkpoint inhibition (3, 4). However, this principle should not be generalized mechanically across all hepatobiliary and pancreatic tumors. Each combination should be matched to a defined biological defect, such as vascular dysfunction, stromal exclusion, deficient priming, or myeloid dominance.

## 5 Biomarkers must move beyond PD-L1

A microenvironment-centered model requires a broader mechanistic biomarker strategy. PD-L1 expression alone is inadequate for most hepatobiliary and pancreatic cancers. Future biomarker development should integrate tumor-intrinsic features, host factors, and spatially resolved microenvironmental information. Relevant domains include MSI/dMMR status, tumor mutational burden, immune gene-expression signatures, T-cell localization, myeloid and fibroblast states, vascular phenotypes, etiology-specific HCC biology, treatment-induced immune remodeling, and spatial immune architecture (20, 21, 28–31). Spatial features are particularly important because immune-cell presence does not guarantee productive antitumor immunity. Effector cells located at the invasive margin, trapped in stromal compartments, or physically separated from malignant glands may have different clinical implications from intratumoral cytotoxic infiltration. In BTC, emerging spatial analyses suggest that tumor-infiltrating lymphocyte distribution may carry predictive information for anti-PD-1 therapy, supporting topology-aware biomarkers rather than bulk immune measurements alone (30). **Table 1** links each proposed barrier domain to candidate mechanistic readouts for prospective translational trials.

**Table 1:** Candidate mechanistic biomarker domains for mapping immune-resistance barriers in hepatobiliary and pancreatic cancers.

Barrier or domain	Candidate diagnostic signature/biomarker readouts	Mechanistic interpretation and therapeutic implication
Impaired antigen presentation and T-cell priming	Low or lost HLA-I/B2M and antigen-processing genes (TAP1/2, PSMB8/9); defective IFN-gamma signaling genes (JAK1/2, STAT1, IRF1); low cDC1 markers (BATF3, CLEC9A, XCR1, IRF8); low CXCL9/CXCL10 and sparse intratumoral CD8^+^ T cells. In HCC, CTNNB1/WNT activation with reduced CCL5/cDC1 recruitment is a practical warning signal (22, 23, 32–35).	Few tumor-reactive effectors are generated or maintained; checkpoint blockade has limited substrate. Favors priming approaches such as radiation, locoregional therapy, vaccines, oncolytic or innate-immune agonists, or strategies that restore antigen presentation.
Trafficking failure and immune exclusion	Spatial IHC/multiplex imaging showing CD8^+^ cells retained at the invasive margin or stromal compartment rather than tumor nests; high CAF/ECM/TGF-β/CXCL12 programs (FAP, ACTA2, COL1A1, TGFB1, CXCL12); abnormal vascular or angiogenic signatures (VEGFA, ANGPT2) (25–27, 30, 31).	Effector cells exist but cannot access malignant cells. Favors immune-access strategies, including vascular normalization, stromal modulation, chemokine-axis targeting, or combinations designed to improve tumor-nest penetration.
Suppressive cellular networks	High TAM/MDSC/Treg/CAF burden: CD68, CD163, MRC1, TREM2, SPP1, CSF1R; ARG1, S100A8/A9, CXCR2; FOXP3, CTLA4, CCR8; FAP/PDGFRB and immunosuppressive cytokines such as IL10, TGFB1, CSF1, and CXCL8 (11–17, 25, 29, 36).	Antitumor immunity is actively disabled by myeloid, regulatory, and fibroblast networks. Favors macrophage or MDSC targeting, Treg modulation, CAF reprogramming, and rational checkpoint combinations beyond PD-1/PD-L1 alone.
Metabolic and vascular dysfunction	Hypoxia and poor perfusion markers (HIF1A, CA9, VEGFA), lactate/glycolysis markers (LDHA, SLC16A3/MCT4), adenosine pathway markers (ENTPD1/CD39, NT5E/CD73), nutrient-depletion pathways (IDO1, ARG1), and functional imaging or circulating markers of hypoxia/perfusion (3, 4, 15–17, 36).	T cells are metabolically impaired or physically starved of oxygen and nutrients. Favors vascular normalization, anti-VEGF-containing combinations, metabolic remodeling, or adenosine/IDO/arginase-axis strategies in biomarker-selected settings.
Additional host-factor domain: gut-liver/biliary axis	Stool metagenomics/16S and fecal metabolomics; microbial β-diversity; bile-acid profiles; gut permeability/endotoxin markers; HCC-associated taxa or species linked to ICI outcomes, including Bacteroides, Bacteroides thetaiotaomicron, Collinsella, Ruminococcus, and Veillonella signals reported in recent studies (37–39).	Host microbial and bile-acid signals can shape dendritic-cell, macrophage, and T-cell tone upstream of the local TME. These readouts are not stand-alone companion diagnostics but may help stratify baseline immune fitness and guide microbiome-informed interventions.
Cross-cutting tumor-cell-intrinsic resistance	Loss-of-function or escape alterations in JAK1/2, B2M/HLA-I, antigen-processing machinery, CTNNB1/WNT-β-catenin activation, MYC-driven immune-evasion programs, and other oncogenic or epigenetic states (32–36, 40).	PD-1/PD-L1 failure can be hardwired into cancer cells and may persist even when the surrounding TME is permissive. These alterations should be assessed in parallel with TME barriers before escalating checkpoint blockade.
Mechanism-guided combination strategy and trial design	Baseline barrier assignment plus on-treatment confirmation of reversal: antigen-release/IFN-gamma/cDC1 induction, CD8+ tumor-nest penetration, myeloid or CAF-state shifts, perfusion/hypoxia/metabolic markers, and early ctDNA dynamics. Examples include TACE plus PD-(L)1/VEGF or VEGFR-directed therapy in HCC and CD40 agonism plus chemotherapy in PDAC (41–43).	The added agent should correct a defined bottleneck, and the trial should verify that correction before claiming synergy. Favors biomarker-enriched umbrella or platform designs with paired tissue, spatial, imaging, or blood-based pharmacodynamic readouts when feasible.

Two caveats are important when using this barrier map. First, immune resistance is not exclusively microenvironmental. PD-1/PD-L1 failure can also be “hardwired” into cancer cells through tumor-cell-intrinsic pathways that blunt immune recognition despite apparently adequate checkpoint blockade. Loss-of-function alterations in JAK1/2 can disable interferon-gamma responsiveness, while B2M or HLA-I loss can impair antigen presentation and T-cell recognition (32, 33). In HCC, WNT/β-catenin activation is a well-described immune-exclusion program that reduces dendritic-cell recruitment and CD8^+^ T-cell infiltration and can contribute to both tyrosine kinase inhibitor and immune-checkpoint inhibitor resistance (34, 35); targeting MMP9 in CTNNB1-mutant HCC has also been shown to restore CD8^+^ T-cell-mediated antitumor immunity and improve anti-PD-1 efficacy in preclinical and translational models (40). MYC provides another tumor-intrinsic route to immune escape by regulating antigen presentation, checkpoint ligands, myeloid recruitment, and immune-metabolic programs (36). These genomic and transcriptional states should therefore be measured alongside TME features rather than treated as downstream consequences of the TME.

Second, host-factor domains can sit upstream of the tumor microenvironment. The gut-liver and gut-biliary-liver axes are especially relevant in HCC and BTC because portal venous flow, bile-acid cycling, microbial metabolites, and barrier permeability continuously expose the liver and biliary tract to intestinal signals. Gut dysbiosis may alter dendritic-cell maturation, macrophage polarization, T-cell priming, bile-acid signaling, and systemic inflammatory tone, thereby modulating whether a tumor becomes permissive or resistant to immunotherapy. Recent HCC studies support this concept: baseline stool microbial features have been associated with clinical response and survival after anti-PD-1-based therapy, and Bacteroides thetaiotaomicron has been reported to enhance anti-PD-1 efficacy by reprogramming dendritic cells through the KLF2/TLR9 axis (37–39). In BTC, the gut-biliary-liver axis is less mature as a predictive biomarker, but it provides a plausible host-factor layer that may interact with cholestasis, bile-acid metabolism, chronic inflammation, and macrophage-rich stromal biology (38).

The goal should not be to identify one universal biomarker. Instead, the field should define which barrier or host/tumor-intrinsic domain is therapeutically dominant in a given patient. A tumor with poor dendritic-cell priming may require a different combination from a tumor with abundant T cells trapped outside malignant glands. A macrophage-dominant BTC may require a different strategy from a VEGF-driven HCC. A fibrotic, hypoxic PDAC should not be treated as though PD-1 signaling were the only missing target, and a JAK1/2-, B2M-, CTNNB1-, or MYC-driven immune-evasion state should not be mistaken for a purely reversible TME defect. Spatial pathology, multiplex immunohistochemistry, transcriptomics, tumor genomic profiling, microbiome/metabolite studies, and longitudinal blood-based monitoring may help move clinical trial design from empiric combination therapy toward mechanism-guided immunotherapy.

Mechanism-guided translation requires a prespecified barrier and proof of reversal. EMERALD-1 and LEAP-012 illustrate TACE plus PD-(L)1 blockade with VEGF/VEGFR targeting in HCC (41, 42). OPTIMIZE-1 tests CD40 agonism plus mFOLFIRINOX as priming/myeloid reprogramming in PDAC, but remains single-arm (43).

## 6 Discussion

Checkpoint blockade has delivered real progress in HCC and BTC, but it has also exposed the limits of a checkpoint-centered model. In hepatobiliary and pancreatic cancers, immune resistance is often determined less by the presence of PD-1/PD-L1 signaling than by the broader architecture of the tumor microenvironment, the tumor-cell-intrinsic capacity to respond to interferon and present antigen, and host-factor inputs such as the gut-liver and gut-biliary-liver axes. HCC, BTC, and PDAC should therefore be viewed not merely as tumors with different response rates to ICIs, but as malignancies with distinct dominant barriers within a shared ecosystem of immune dysfunction.

Three testable hypotheses follow: biomarker-unselected triplets will remain inefficient; treatment sequence should generate, admit, and reinvigorate effectors in order; and negative trials should be analyzed as failures of barrier reversal or compensatory switching, not simply as immunotherapy failures.

The next generation of immunotherapy trials should be designed around these barriers. Rather than repeatedly adding checkpoint inhibitors to unselected populations, future strategies should ask whether a tumor needs immune priming, improved T-cell access, myeloid or fibroblast reprogramming, vascular normalization, metabolic remodeling, correction of tumor-intrinsic immune invisibility, host-factor modulation, or biomarker-selected combinations. Reframing immune resistance in this way may help convert biologically plausible combinations into clinically meaningful and durable benefit.
